# Familial Diabetes

**Published:** 1914-05

**Authors:** 


Familial Diabetes. F. A. Long of Madison, Neb., reports in the *Western Med. Rev.*, January, 1914, the following instances: 1, two brothers; 2, father and two sons; 3, husband and wife and two daughters; 4, the sister of the man in 3; her two daughters, one son and a granddaughter. All were farmers, none of neurotic or rheumatic tpye.

